# Mucosal and Cutaneous Human Papillomavirus Infections and Cancer Biology

**DOI:** 10.3389/fonc.2019.00355

**Published:** 2019-05-08

**Authors:** Tarik Gheit

**Affiliations:** Infections and Cancer Biology Group, International Agency for Research on Cancer (IARC), Lyon, France

**Keywords:** Papillomavirus, infection and cancer, transformation, anogenital cancer, skin cancer

## Abstract

Papillomaviridae is a family of small non-enveloped icosahedral viruses with double-stranded circular DNA. More than 200 different human papillomaviruses (HPVs) have been listed so far. Based on epidemiological data, a subgroup of alphapapillomaviruses (alpha HPVs) was referred to as high-risk (HR) HPV types. HR HPVs are the etiological agents of anogenital cancer and a subset of head and neck cancers. The cutaneous HPV types, mainly from beta and gamma genera, are widely present on the surface of the skin in the general population. However, there is growing evidence of an etiological role of betapapillomaviruses (beta HPVs) in non-melanoma skin cancer (NMSC), together with ultraviolet (UV) radiation. Studies performed on mucosal HR HPV types, such as 16 and 18, showed that both oncoproteins E6 and E7 play a key role in cervical cancer by altering pathways involved in the host immune response to establish a persistent infection and by promoting cellular transformation. Continuous expression of E6 and E7 of mucosal HR HPV types is essential to initiate and to maintain the cellular transformation process, whereas expression of E6 and E7 of cutaneous HPV types is not required for the maintenance of the skin cancer phenotype. Beta HPV types appear to play a role in the initiation of skin carcinogenesis, by exacerbating the accumulation of UV radiation-induced DNA breaks and somatic mutations (the hit-and-run mechanism), and they would therefore act as facilitators rather than direct actors in NMSC. In this review, the natural history of HPV infection and the transforming properties of various HPV genera will be described, with a particular focus on describing the state of knowledge about the role of cutaneous HPV types in NMSC.

## Introduction

Papillomaviridae is a family of small non-enveloped icosahedral viruses with double-stranded circular DNA, which range in length from 5,748 bp for *Sparus aurata* papillomavirus 1 (SaPV1) to 8,607 bp for canine papillomavirus type 1 (CPV1). Papillomaviruses (PVs) infect basal keratinocytes of the mucosal and cutaneous epithelia of both animals (reptiles, birds, marsupials, and others) and humans (1). In 2016, the first characterization of a PV in fish (SaPV1) rendered this family of viruses much older than expected, with an emergence 450 million years ago (2). PVs are considered to be host-restricted; however, in rare cases cross-species transmission may also occur (3, 4).

Based on the nucleotide sequences of the major capsid protein L1, the PV study group within the International Committee on Taxonomy of Viruses (http://ictv.global/report/papillomaviridae) has classified the PVs into 53 genera, among which only 5 genera include PVs that infect humans (HPVs). To be classified as a novel PV type, the nucleotide sequence of L1 must share < 90% similarity with other PVs (5). During the past decade, there has been an exponential increase in the identification of new betapapillomaviruses (beta HPVs) and gammapapillomaviruses (gamma HPVs) that have been discovered with the advent of new technologies such as next-generation sequencing. More than 200 different HPV types have been listed by the International HPV Reference Center (www.hpvcenter.se) (6), and this number continues to expand (7).

HPV types are organized into five major genera: alpha, beta, gamma, mu, and nu (5). The genus gamma includes the majority of the known HPVs, with 99 types, followed by the genera alpha (*n* = 65) and beta (*n* = 54). The genera mu and nu include only 3 and 1 types, respectively (www.hpvcenter.se, on 2019-01-30). A recent study reported the identification of the complete genome of 83 previously unknown HPV types, among which 69 were classified as gamma (8).

Based on epidemiological and biological data, a subgroup of 12 mucosal HPV types classified in the genus *Alphapapillomavirus* (alpha HPV), i.e., HPV16, 18, 31, 33, 35, 39, 45, 51, 52, 56, 58, and 59, referred to as high-risk (HR) HPV types, has been classified as carcinogenic (IARC Group 1), and eight other HPV types, i.e., HPV26, 53, 66, 67, 68, 70, 73, and 82, have been classified as probably or possibly carcinogenic (IARC Groups 2A and 2B) (9, 10). The HR HPV types are the etiological agents of several cancers, such as those of the cervix, vagina, vulva, anus, penis, and a subset of head and neck cancers (HNCs), particularly oropharyngeal cancer (11). In 2012, 15.4% of cancers worldwide were attributed to carcinogenic infections (12). Together, the HPV-related cancers represent 630,000 new cancer cases per year, which account for ~30% of all cancers induced by infectious agents (10). Epidemiological and biological studies showed that HPV16 is the most oncogenic of the HR HPVs. The genus alpha also includes low-risk (LR) HPV types that cause benign lesions. HPV6 and 11, the most studied LR HPV types, induce benign genital warts or condylomata acuminata (13). These HPV types are also found in recurrent respiratory papillomatosis (14), in children. The genus alpha also includes a few cutaneous HPV types (HPV2, 3, 7, 10, 27, 28, and 57), which cause common and plantar warts (15–18).

The genera beta, gamma, mu, and nu contain HPVs that infect cutaneous epithelia. The genus alpha also contains some HPV types, such as HPV2, 3, and 10, that colonize the human skin. The mu and nu genera include only a few HPV types, whereas the genus beta includes more than 54 HPV types, which are subdivided into five species (β1–5), and the genus gamma includes 98 types subdivided into 27 species (6). In the immunocompetent population, these so-called cutaneous HPV types induce asymptomatic chronic infections. However, members of the genera gamma, mu, and nu can induce benign skin lesions, e.g., cutaneous papillomas or warts (5). In addition, there is growing evidence of an etiological role of beta HPVs in non-melanoma skin cancer (NMSC), most likely in association with ultraviolet (UV) radiation [reviewed in (19–21)].

In this review, we describe the natural history of mucosal and cutaneous HPV infections, and discuss the transforming properties of a subset of them that have been shown or are suspected to play a causative role in various human cancers. In particular, we focus on describing the state of knowledge about the role of cutaneous HPV types in NMSC, because many existing reviews already cover all the aspects of mucosal HPV type infection and its role in transformation.

## Genomic Organization of HPV and Viral Gene Products

**Figure 1:**
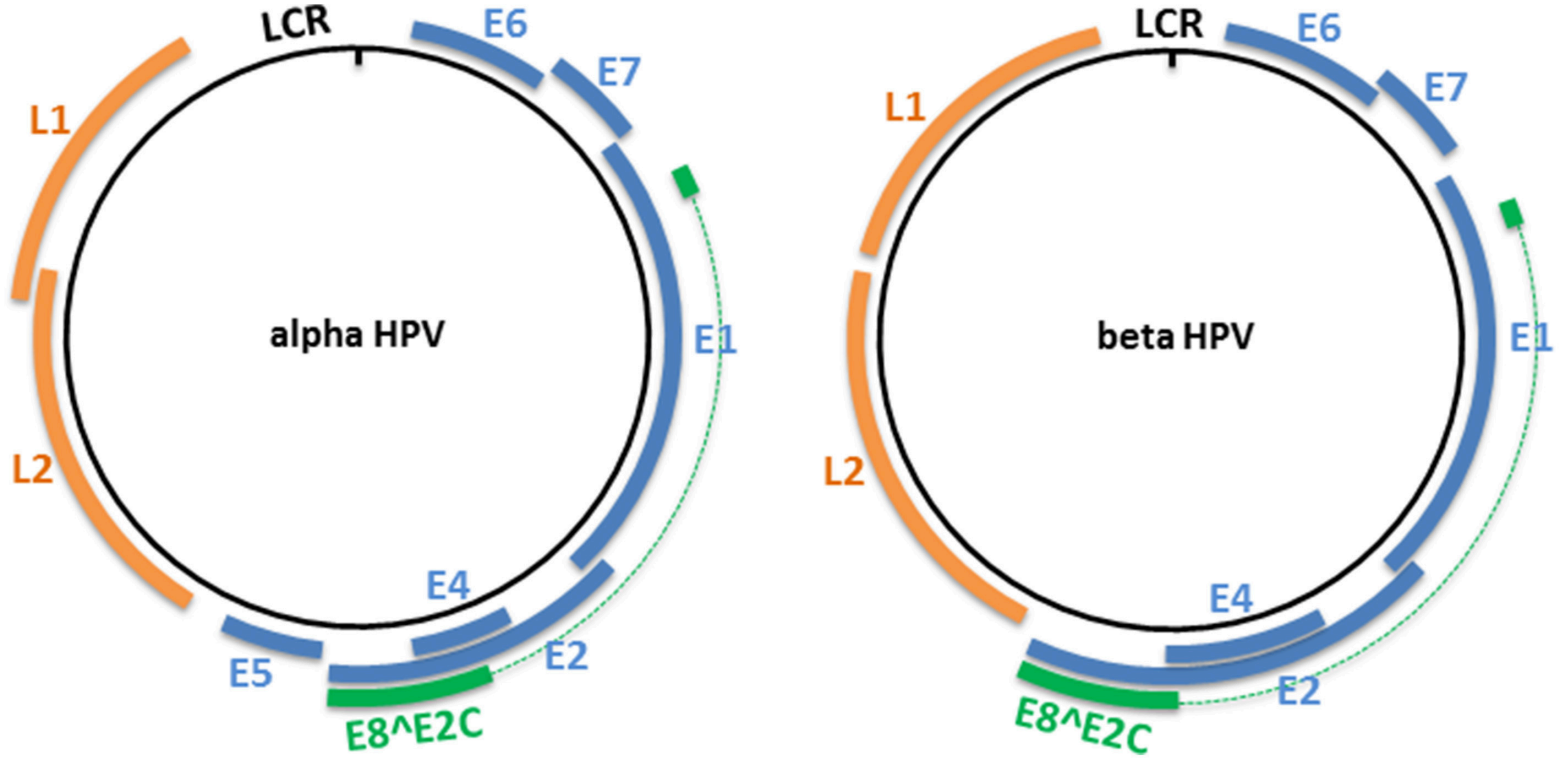
Genome organization of alpha and beta HPV types. The circular dsDNA genome of high-risk alpha and beta HPV types is represented by a black circle. The positions of the early and late genes are represented by blue and orange, respectively. Both represented PV genera have the potential to express an E8^∧^E2C transcript that encodes for a protein that includes an E8 domain fused to the hinge and DNA-binding domains of E2. LCR, long control region.

The HPV genome is organized into three major regions: (i) a long control region (LCR), also called the upstream regulatory region (URR), located between the L1 and E6 open reading frames (ORFs), which contains the early promoter and regulatory element involved in viral DNA replication and transcription; (ii) the early region, which encodes the E1, E2, E4, E5, E6, and E7 proteins involved in viral gene expression, replication, and survival; and (iii) the late region, which encodes the structural proteins (L1 and L2). This organization is shared among all alpha HPVs (Figure 1). However, only four ORFs (those of E1, E2, L1, and L2) are necessary to fulfill the requirements to ensure the viral replication and shedding of the virus, and are present in all known PVs (22). Certain HPV genera and types lack an ORF. For example, the E5 ORF is lacking from HPV types that belong to the genera beta, gamma, and mu, and both the E5 and E6 ORFs are lacking from three gamma HPV types (HPV101, 103, and 108) (23, 24). LR HPV types (i.e. HPV6 and 11) encode for two E5-like proteins E5γ and E5δ (25). Beta and gamma HPV types have the particularity of harboring a shorter LCR compared with members of other genera (Figure 1).

All PVs also have the potential to express an E8^∧^E2C transcript (or equivalent), encoding for a protein that includes the E8 domain fused to the hinge and DNA-binding domains of E2 (26). This protein acts as a transcriptional repressor, and represses E1/E2-dependent replication of the viral origin (27–29).

## Natural History of Mucosal HPV Infections

Mucosal HPV infections occur during the first sexual exposures in early adulthood (30), although non-sexual infection may also be possible (31). Although a majority of sexually active women will acquire a genital HPV infection, most (>90%) cervical HPV infections are resolved by the host immune system within 1–2 years (32, 33) and give rise only to asymptomatic infections. However, a minority of HPV infections become persistent. The risk of developing epithelial cell abnormalities and cancer is then increased (32). Persistent infection may be explained by several factors. Alcohol consumption and the HR HPV load synergistically increased the risk of persistent HR HPV infection in women (34). Cigarette smoking alone also plays an important role in the acquisition of persistent HPV infections in women, by decreasing the probability of clearing oncogenic infections (35). Smoking is also a risk factor for a persistent oral HPV infection (36). Host genetic risk factors may also predispose an individual to persistent HPV infection and developing cervical cancer, as supported by the high rate of heritability of cervical cancer (37–39). Defective immune response due to genetic variations (e.g., inflammasome genetics) has been associated with virus persistence and progression to cervical cancer (39–41). Human leukocyte antigen (HLA) genes also play a key role in the persistence of HPV infections and progression to cervical cancer, depending on their ability to bind to HPV antigens (39, 42).

Many natural HPV intra-type variants that differ from the prototypes have been identified, and some of them have been associated with the persistence and clinical outcome of cervical HPV infections (43–46). Several studies showed that EUR-350 T and EUR-350 G, which are HPV16 E6 variants, could influence viral persistence (47, 48). Recently, Mirabello et al. (49) evaluated a large collection of 5,570 HPV16-infected case–control samples using HPV whole-genome sequencing, and showed that E7 variation from the prototype sequence greatly decreases the risk of invasive cancer. That study also highlighted the presence of rarely detected variants of HPV16 that are consistent with the antiviral activity of the human APOBEC3 cytidine deaminase family. Moreover, APOBEC3A is stabilized in human keratinocytes by HR HPV E7, but not by E7 of LR HPV types (50).

The HR mucosal HPV genome integrates into host chromosomes in anogenital and oropharyngeal cancers, and it is considered an important driver of carcinogenesis.

There is not yet consensus on whether this is an early or a late event in carcinogenesis (51, 52). The integration occurs frequently within the E1 or E2 proteins, leading to dysregulation of expression of the oncoproteins E6 and E7 due to loss of the E2 repression function (53). The stability of E6 and E7 mRNAs is increased when HPV16 DNA is integrated into the human genome (54).

The frequency of viral integration increases with the severity of the cervical precancerous lesions. Integration of HPV into the host genome is reported in a majority of cervical cancers (83%) (55), and HPV18 has been found integrated into almost all cervical cancers (56, 57). HPV16 can be present as episomal or integrated forms, or both (58).

The DNA integration event could contribute to the HPV-driven malignant transformation of cervical cells, by affecting the expression of key host genes near the integration site (59). In addition, the existence of long-distance regulation among the integrated HPV fragment, the MYC gene, and the 8q24.22 region has been reported (60).

Although it was commonly accepted that integration occurred randomly into the host genome, several studies have shown that non-random HPV integration occurs. Integration of HPV16 sequences appears to take place preferentially in common fragile sites in human chromosomes (61, 62), in transcriptionally active regions (63), or near microRNAs (64).

Carcinogenesis can also be enhanced by epigenetic events. In cervical cancer, HPV16 E2 binding sites (E2 BS1 and E2 BS2), which play a role in transcriptional repression of E6 and E7 oncoproteins, are heavily methylated. The binding of E2 protein on these sites is then prevented, which leads to the upregulation of E6 and E7 oncoproteins (65). Host or viral epigenetic changes may be enhanced by the integration of HPV. A recent study showed that L1 gene methylation increases significantly according to the grade of the cervical lesion, and a high methylation rate of this gene correlates with the physical status of HPV integration (66, 67).

Data on HPV integration in HNC are still sparse and contradictory. Parfenov et al. (68) reported that 25 out of 35 HNCs showed integration of HPV into the human genome. Other studies reported HPV integration rates of 39% in oral squamous cell carcinoma (SCC) (69) and 43% in HNC (70), which is lower compared with HPV-positive cervical cancer. Episomal, integrated, and mixed forms of HPV are found in HPV-positive HNC. Moreover, a recent study, based on analysis of the Cancer Genome Atlas sequencing data, reported for the first time the presence of viral–human hybrid episomes in HPV-positive HNC (71).

## Natural History of Cutaneous HPV Infections and Clinical Implications

Cutaneous HPV types are ubiquitous and are widespread in the general population. Up to 90% of healthy individuals tested positive for beta HPV types (72–74). The infection occurs in young children through skin-to-skin contact (75, 76). The cutaneous HPVs have been proposed to infect hair follicle stem cells of healthy individuals, where they constitute a reservoir of persistent infection (77, 78). DNA from cutaneous HPVs is frequently detected in hair bulbs, independently of the anatomical region where the hairs were plucked (79). Schmitt et al. (80) showed that keratinocyte stem cells (KSCs) in rabbit hair follicles co-localize with the primary target cells of cottontail rabbit papillomavirus (CRPV). They showed presence of CRPV early transcripts in clonogenic cells of the hair follicles soon after infection, suggesting that hair follicle stem cells are the initial target cells for CRPV (80), which was shown to induce cutaneous warts and carcinomas (81).

Epidemiological studies in humans showed that the cutaneous HPV population present in hair follicles mirrors the HPV prevalence in the skin of the same individual, which makes eyebrow hair an excellent sampling method to characterize the individual cutaneous HPV population (79, 82). Studies on intra-familial transmission showed that similar spectra of beta HPV types are present within members of the same family. Babies and their parents share some of the beta HPV types (83), which can persist for many years on healthy skin (84, 85). Transmission of beta and gamma HPVs has been demonstrated in couples (86).

Exposure to cutaneous HPV is common. Serological studies measuring antibodies against the type-specific L1 major capsid protein of HPV showed that 52% of the Dutch population and 67% of the Italian population were exposed to beta HPV infections (74). A recent seroprevalence study, based on ten beta HPV types, showed that 39% of healthy men were seropositive for at least one beta HPV type (87). Seroconversion for beta and gamma HPV types appears to be slow and to increase with age (88). Low viral loads in immunocompetent individuals (89) and continuous renewal of the infected keratinocytes may explain why only half of the infected individuals develop antibodies against cutaneous HPV types (90, 91).

Cutaneous HPVs are highly prevalent in the rare hereditary disease epidermodysplasia verruciformis (EV), which tends to progress to cutaneous SCC (cSCC), frequently located at sun-exposed anatomical sites (92). HPV5 and 8, two members of species β1, were isolated from cSCC in patients with EV (93). These two types were classified as possibly carcinogenic to humans (IARC Group 2B) (10).

In organ transplant recipients, a 65–250-fold increased risk of developing SCC compared with the general population was reported (94, 95). In addition, in HIV-positive individuals, several studies reported at least a 2-fold increased risk of cSCC compared with HIV-uninfected people (96, 97). The correlation between the immunodeficiency state and an increased risk of developing NMSC suggested a possible role of infectious agents (98), such as cutaneous HPV.

This observation is corroborated by growing evidence showing an association between cutaneous infection and the risk of developing NMSC in immunocompetent individuals under certain conditions (e.g., UV radiation exposure) [reviewed in (21, 99)], in particular in cSCC (100–102).

Epidemiological and biological data suggested that beta HPV types, and species β1 and β2 in particular, may be linked to cSCC development in immunocompetent individuals (99, 100) [reviewed in (21)].

Interestingly, the cutaneous HPV viral load was higher in actinic keratosis, which is considered to be the precursor lesion of cSCC, compared with cSCC (< 1 copy per cell), suggesting a possible role of cutaneous HPV types in the initiation of skin carcinogenesis but not in the maintenance of the cancer phenotype, by exacerbating the accumulation of UV radiation-induced DNA breaks and somatic mutations (the hit-and-run mechanism) (103, 104). The absence of HPV mRNA (105) and the lack of evidence for the integration event in skin tumors support this scenario.

The *Mastomys natalensis* papillomavirus (MnPV)-infected rodent model *Mastomys coucha* was previously described and used to evaluate the role of PVs in NMSC. In these mice, which can be naturally infected by MnPV, skin lesions such as papillomas or keratoacanthomas can be induced (106). Using this model, Hasche et al. (107) clearly demonstrated cooperation between UVB radiation exposure and MnPV infection in the first step of NMSC initiation. MnPV-positive animals chronically exposed to UVB radiation developed lesions significantly more frequently than MnPV-negative animals (107). Moreover, the authors showed higher MnPV viral loads in well-differentiated and keratinizing SCCs (KSCCs) compared with normal skin. Of note, these KSCC lesions share histological similarities to human SCC pre-malignant lesions, such as actinic keratosis, that normally show higher cutaneous HPV DNA loads than SCC, as discussed above. In addition, non-keratinizing SCCs (nKSCCs) occurring in mice UV-irradiated at higher doses showed low levels of (or no) viral DNA and often harbored p53 mutations, again mirroring the evolution of SCC in humans. The loss of viral DNA and viral gene expression in nKSCC lesions is most likely due to their undifferentiated state, which no longer sustains the infection. However, despite the lack of viral DNA, animals with nKSCCs developed antibody response to MnPV capsids, which clearly highlights past exposure to the virus, similar to the situation observed in SCC cases (108). Therefore, the *Mastomys* model shows parallels with a natural infection by cutaneous HPV and provides a good model to study the association between cutaneous HPV infection, UV radiation and SCC. Most importantly, this model provides further evidence for a hit-and-run mechanism.

Many independent studies reported the presence of beta HPV types at different anatomical regions other than the skin, such as the oral mucosal epithelium, genital sites, and the anal canal (109–112), or investigated the role of cutaneous HPV in various malignancies (e.g., male external lesions, breast cancers, salivary gland tumors, esophageal cancer) other than skin cancer, without showing any association (113–116). However, a recent study reported an increased risk of HNC in individuals who tested positive for HPV5 of species β1, as well as other HPV types of species γ11 and γ12 (117). The same trend was reported in another study, in which β1 HPV5 and β2 HPV122 were significantly associated with HNC (118).

Whereas, beta HPVs are known to infect cutaneous tissues, epidemiological data showed that β3 HPV types are also present in the mucosal epithelium, suggesting a dual tropism (111, 119). Moreover, this species shares biological similarities with mucosal HR HPV types such as HPV16 in *in vitro* and *in vivo* experimental models (120, 121). A study showed that β3 HPV49 transgenic mice, after 4-nitroquinoline 1-oxide treatment, were prone to develop upper digestive tract tumors (121).

Epidemiological and biological data on the role of gamma HPV types are sparse, and do not support an etiological role in NMSC. However, this genus includes an increasing number of members that may deserve more investigation. Using deep sequencing, a member of species γ27 (HPV197) has been isolated exclusively from skin cancers. However, additional studies are required to demonstrate an etiological link between this type and skin cancer (122, 123).

## Entry and Life Cycle of HPV

The viral particle reaches the basal layer of the epithelium via micro-wounds or micro-fissures, or via hair follicles (80, 124, 125), to infect basal keratinocytes or stem epithelial cells. In the cervix, HR HPV may directly reach the single layer of cuboidal epithelial cells from the squamocolumnar junction located between the endocervix and the ectocervix (126); this anatomical region is therefore more sensitive to infection. In the oral cavity, HPV infection is believed to occur in tonsillar crypts, where a comparable cellular structure exists (127).

The infectious entry of the virus into the cell is intimately linked to proliferating cells of the regenerating basal epithelium. Broniarczyk et al. (128) showed that the viral particle can remain infectious after several weeks on the surface of senescent cells, which are resistant to HPV infection; however, reactivation of the cell cycle by p53 siRNA led to the entry of the virus (128).

**Figure 2:**
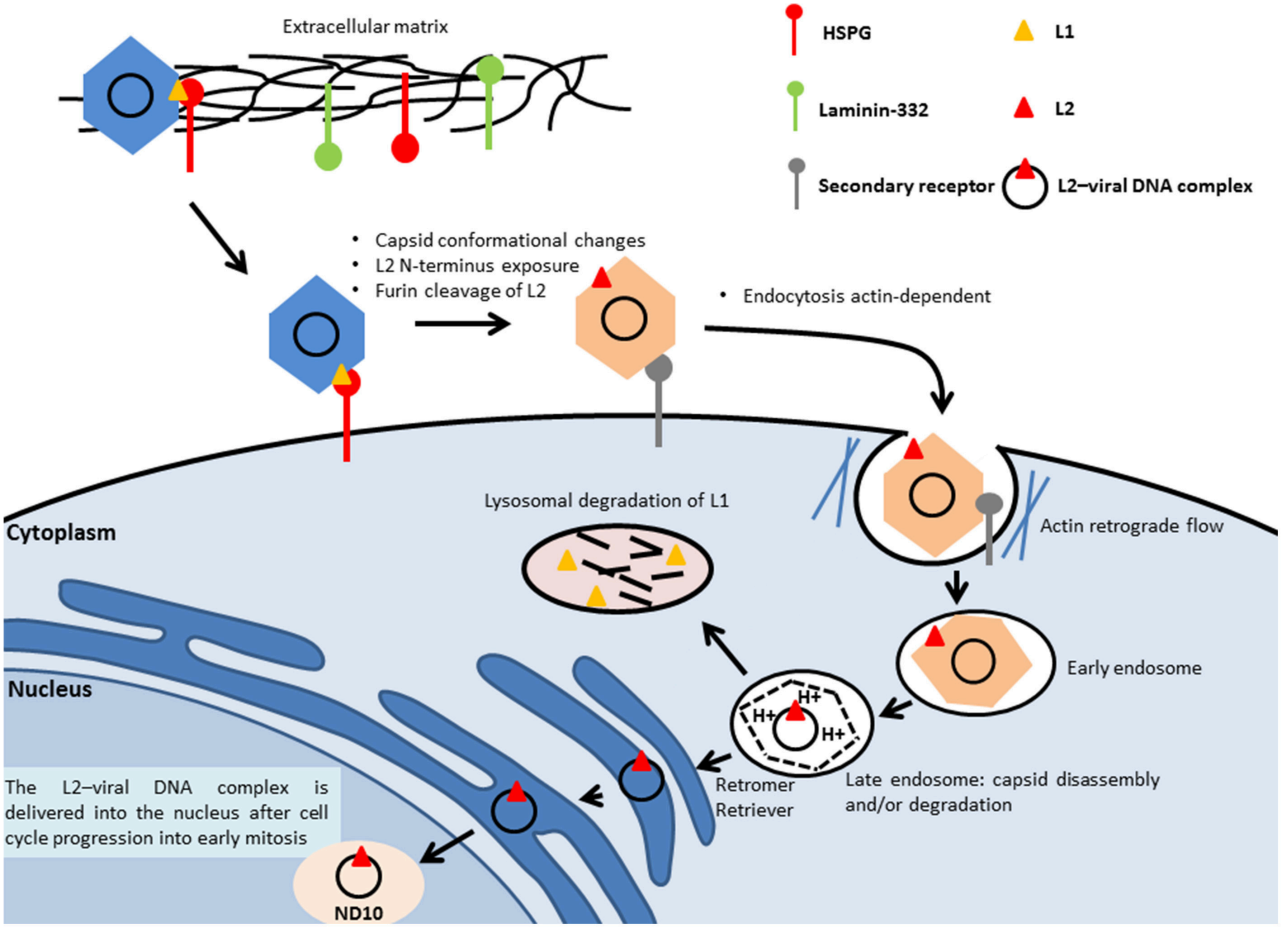
Entry of HPV. The virus binds to the heparan sulfate chains of proteoglycans (HSPGs) located on the cell membrane or on the extracellular matrix through the major capsid protein L1, which leads to the conformational change of the capsid and the subsequent L2 externalization on the surface of the viral particle. Next, the N-terminus of the minor capsid protein L2 is cleaved at a conserved furin cleavage consensus site, which facilitates the binding of HPV to an unidentified secondary receptor, allowing subsequent internalization that is actin-dependent. The L2–viral DNA complex and a portion of L1 are delivered to the trans-Golgi network, through retromer- and retriever-mediated vesicular trafficking. Inside the late endosomes, the capsid of the viral particle is disassembled in a low-pH environment, leading to the dissociation of the major capsid protein L1 from the minor capsid L2, mediated by host-cell cyclophilins. The L2–viral DNA complex is then delivered into the nucleus to the nuclear domain (ND10), during cell mitosis.

To initiate the infection, the virus binds to the heparan sulfate chains of proteoglycans (HSPGs) located on the cell membrane or on the extracellular matrix (ECM) through the major capsid protein L1, leading to cyclophilin (CyP) B-mediated conformational changes of the capsid structure, which expose the minor capsid protein L2 on the surface of the viral particle (Figure 2). The cleavage of L1 in the extracellular space by a serine protease, kallikrein-8, appears to be crucial for the efficient externalization of L2 (129). On the ECM, the virus can also bind to a transient binding receptor, laminin-322 (formerly called laminin-5) (130).

Next, the N-terminus of the minor capsid protein L2 is cleaved with a CyP-independent mechanism at a conserved furin cleavage consensus site to expose the L2 amino acids 17–36 (the RG1 neutralizing epitope), which appears to be important for the interaction of the capsid with an unidentified secondary receptor (131, 132). The binding of HPV to the secondary receptor allows subsequent internalization.

Using HPV16 pseudovirion (PsV) particles covalently labeled with some fluorophores Schelhaas et al. (133) showed that HPV16 PsVs moved along the outside cell membrane in correspondence to the actin protrusions and were transported inside the cells by actin retrograde flow. Upon inhibition of the actin flow by blebbistatin, the virus particles could not be actively transported along actin-rich protrusions, which resulted in reduced efficiency but not in complete abolishment of the infection (133). In a more recent work, using different compound inhibitors and siRNAs, Schelhaas et al. (134) showed that the entry of HPV16 into HeLA and HaCaT cells was actin-dependent and clathrin-, AP2-, caveolin-, flotillin-, and dynamin-2-independent (134). Although the exact mechanism of membrane protrusion/vesicle formation during virus endocytosis remains unclear, Schelhaas's study clearly showed that actin polymerization and depolymerization are crucial for HPV16 endocytosis, in particular for scission of endocytic vesicles (134). Once inside the cell, the virus needs to reach the replication machinery within the nucleus (Figure 2). The virus is delivered to an early endosome or macropinosome-like endosome that matures into a late endosome [reviewed in (135)]. Inside the late endosomes, the capsid of the viral particle is disassembled in a low-pH environment, leading to the dissociation of the major capsid protein L1 from the minor capsid L2, mediated by host-cell CyPs (136). However, a recent study showed that residual conformationally intact L1 protein remains in complex with the viral genome (137). The L2–viral DNA complex traffics to the trans-Golgi network, mediated by the retromer complex (138). Retromer is a heteropentameric complex composed of dimer of sorting nexins (SNX1, SNX2, SNX5, and SNX6) and a vacuolar protein sorting (Vps) trimer containing Vps26, Vps29, and Vps35, which play a central role in the retrieval of several different cargo proteins from the endosome to the trans-Golgi network. The trafficking also involves retriever, a multi-protein complex analogous to retromer (139), and involves the cellular adaptor protein SNX17. This complex is associated with endosomes and is essential for the recycling of multiple cargoes. A proteomic approach has shown that SNX17 interacts with HPV16 L2 (140). The observation that the SNX17 binding site is conserved among different PV genera suggests that it has an important role in regulating the PV life cycle and replication. The SNX17/L2 interaction is indeed required for HPV16 infection and appears to play key roles at different stages of the infection, being important for effective capsid disassembly and L1 dissociation. The interaction of L2 with SNX17 also contributes to the maintenance of viral capsids in the late endosomal compartment, by protecting them from lysosomal degradation and allowing L2–DNA complexes to egress from the endosomes (141). A related protein, SNX27, is part of the retromer complex and also interacts with L2, but through its PDZ domain. SNX27 appears to be important for virion trafficking and to cooperate with SNX17 in this event (142).

L2 is required for efficient trafficking of the viral genome to the nucleus (138) and remains associated with the viral DNA when the transport vesicles deliver the L2–viral DNA complex inside the nucleus [reviewed in (143)], most likely together with residual L1.

Delivery of the L2–viral DNA complex into the nucleus requires cell cycle progression into early mitosis (144). Nuclear envelope disruption is also required for nuclear import of the L2–viral DNA complex (145) (Figure 2).

Inside the nucleus, L2 mediates the viral genome delivery to the nuclear domain (ND10) (146, 147), also known as promyelocytic leukemia (PML) bodies, where early viral transcription and replication will take place, as for many DNA viruses. ND10 structures are host restriction factors that limit viral infection as part of the intrinsic defense against viral infection (148). Stepp et al. (149) showed that Sp100 nuclear antigen, one of the components of ND10, represses transcription, replication, and establishment of incoming HPV DNA in the early stages of infection. Upon initial infection, the minor capsid protein L2 leads to the alteration of the composition of ND10 protein, leading to the release or degradation of Sp100 (150). The Daxx protein, another component of ND10, is also recruited (150). It was previously reported that this protein modulates the early gene expression and the transient replication of HPV genomes in U2OS cells (151).

**Figure 3:**
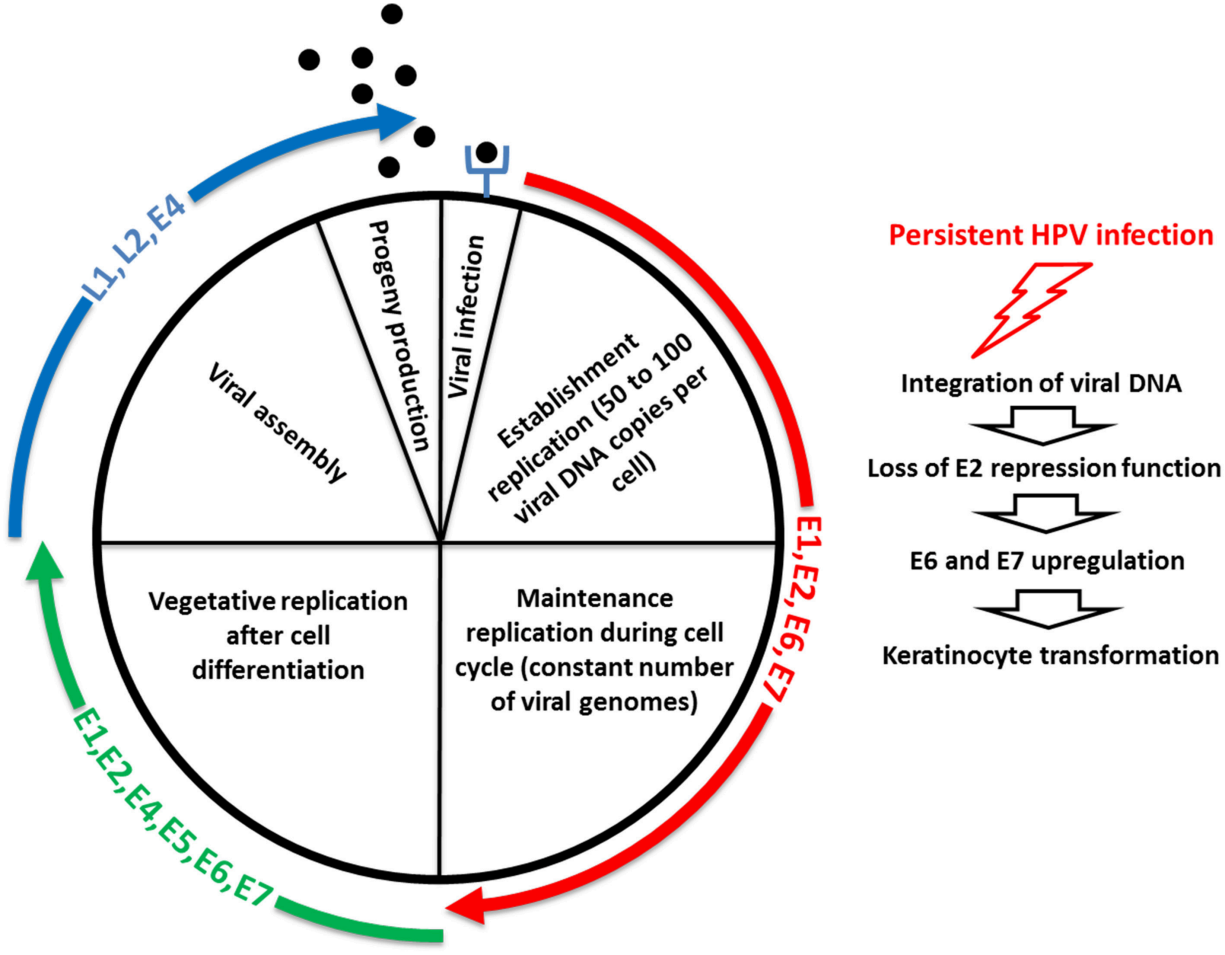
Life cycle of HPV. The first step of the replication cycle of HPV, called “establishment replication,” consists of maintaining a constant number of episomal copies. Viral DNA replication relies on the host DNA replication machinery and is supported by the early viral proteins E1 and E2. After this initial step, the maintenance phase is initiated. This phase consists of creating the conditions to maintain a constant number of viral genomes in the nuclei of undifferentiated basal cells as an extrachromosomal genome to create a persistent infection. In addition to E1 and E2, the oncoproteins E6 and/or E7 are required for stable episomal maintenance of HPV. Upon cell differentiation in stratified epithelium, vegetative or productive viral replication is initiated, with the subsequent production of progeny virions. Here, the oncoproteins E6 and E7 expressed at relatively low levels in differentiated cells play a key role by inactivating tumor suppressor proteins. The activation in differentiated epithelial cells of the late promoter, located in the E7 region, leads to the production of the high levels of E1 and E2 viral proteins required to ensure viral DNA replication. E4 and E5 contribute to efficient productive replication. The capsid proteins L1 and L2 are expressed from the late promoter, and are involved in the encapsidation of newly replicated genomes, resulting in virion release in the superficial layers during desquamation. E4 also plays a role at this step of the viral life cycle, by interacting with the keratin network. The right side of the schematic representation illustrates that a minority of HPV infections become persistent, thus increasing the risk of keratinocyte transformation, through integration of viral DNA into the human genome, which leads to the loss of E2 repression function and subsequent E6 and E7 upregulation.

The replication cycle of HPV is linked to the differentiation of the infected epithelium, and starts with a first step, called “establishment replication,” which consists of maintaining a constant number of episomal copies (50–100 per cell) (Figure 3) [reviewed in (152)]. Viral DNA replication relies on the host DNA replication machinery and is supported by the early viral proteins E1 and E2 (153). Upon nuclear entry, viral DNA replication is initiated by the binding of E2 on specific sites located on the LCR, which is required for the recruitment and binding of E1 helicase to the viral origin of replication (154). The cellular proteins TopBP1 and Brd4 are involved in the initiation of the HPV16 E1/E2-mediated DNA replication (155). Limited viral genome amplification may be obtained by the interaction of NCOR/SMRT repressor complexes with E8^∧^E2C proteins, which inhibits viral replication (156, 157). After this initial step, which aimed to generate a low copy number of genomes, the maintenance phase is initiated (Figure 3) [reviewed in (152)]. This phase consists of creating the conditions to maintain a constant number of viral genomes in the nuclei of undifferentiated basal cells as an extrachromosomal genome to create a persistent infection. The viral genomes need to be correctly segregated during cell division through attachment/tethering of the virus genome to the host-cell chromosome. The transactivation domain at the N-terminal part of E2, with the bromodomain protein Brd4, interacts with the host mitotic chromosomes. Concomitantly, the E2 DNA-binding/dimerization domain binds to E2 binding sites in the LCR of the viral genome (158–162). Brd4 interacts with the E2 transactivation domain of most PVs (163). ChIR1, an ATP-dependent DNA helicase, seems to play a key role by regulating the chromatin association of HPV16 E2, and in maintaining the episomal form of HPV16 (164–166). The structural maintenance of chromosomes (SMC) proteins SMC5 and SMC6 may also play a role in viral genome maintenance by interacting with E2 (167). In addition to E1 and E2, the oncoproteins E6 and/or E7 are required for stable episomal maintenance of HPV (168, 169).

Upon cell differentiation in stratified epithelium, vegetative or productive viral replication is initiated, with the subsequent production of progeny virions (170, 171), [reviewed in (152)] (Figure 3). Uninfected cells leave the basal layer for terminal differentiation, are withdrawn from the cell cycle, and stop the replication of the DNA. Because the amplification of the viral genome requires cellular conditions that enable cell proliferation and thus DNA replication, the virus has developed strategies to prevent cell-cycle arrest and apoptosis signals. Here, the oncoproteins E6 and E7 expressed at relatively low levels in differentiated cells play a key role by inactivating tumor suppressor proteins (e.g., p53, retinoblastoma protein [pRb]) and activating signal transduction, to ensure that the infected cells remain active and progress to the S phase. The activation in differentiated epithelial cells of the late promoter (P670 for HPV16, P811 for HPV18, and P742 for HPV31), located in the E7 region, leads to the production of the high levels of E1 and E2 viral proteins required to ensure viral DNA replication. E4 and E5 contribute to efficient productive replication (172, 173). The capsid proteins L1 and L2 are expressed from the late promoter, and are involved in the encapsidation of newly replicated genomes, resulting in virion release in the superficial layers during desquamation (174) (Figure 3). E4 also plays a role at this step of the viral life cycle, by interacting with the keratin network (175). A more complete description of the function of each of the viral proteins is provided in Table 1.

**Table 1:** Main features and functions of the early and late gene products from alpha and beta HPV types.

**Gene products**	**Alpha HPV features**	**Beta HPV features**
LCR	Also called upstream regulatory region (URR), contains the early promoter and regulatory element involved in viral DNA replication and transcription	
E6	Required for the maintenance of the cancer phenotype Required for stable episomal maintenance	Not required for the maintenance of the cancer phenotype Inhibition of UV radiation-induced damage repair Hampers the differentiation of HPV8-expressing keratinocytes by targeting the PDZ domain-containing protein syntenin 2 Interacts with Notch pathway and promote the transformation process of the infected keratinocytes
	Deregulation of cell cycle Inhibition of apoptosis Cell polarity, migration and attachment The PDZ domain-binding motif of E6 proteins regulates HPV life cycle Upregulation of the hTERT promoter activity	
E7	Required for the maintenance of the cancer phenotype	Not required for the maintenance of the cancer phenotype
	Required for stable episomal maintenance	E7 from HPV38 shows the ability to counteract p53-mediated apoptosis by inducing accumulation of the p73 isoform, ΔNp73
	Deregulation of cell cycle Inhibition of apoptosis	
E5	Not required for the maintenance of the oncogenic phenotype Increases the immortalization effects of HPV16 E6 and E7 Promotes tumor cell motility and cancer metastasis Promotes cell-cycle progression Inhibition of apoptosis	The E5 ORF is lacking
E8^∧^E2	∙ Acts as a transcriptional repressor, and represses E1/E2-dependent replication of the viral origin	
E1	∙ Viral DNA replication	
E2	∙ Ensure the segregation of the viral genome during cell division	
E4	∙ Contributes to efficient productive replication in differentiating cells	
L1	∙ Major capsid protein	
L2	∙ Minor capsid protein	
	∙ L2 is required for an efficient trafficking of the viral genome to the nucleus	
	∙ L2 mediates the viral genome delivery to the nuclear domain (ND10)	

Epigenetic regulation of HPV transcription plays an important role in the virus life cycle. HPV regulatory epigenetic mechanisms have recently been partly elucidated by Pentland et al. (176). The key players in this regulation are the chromatin-organizing CCCTC-binding factor (CTCF) and Yin Yang 1 (YY1), binding, respectively, to the E2 ORF and the viral LCR. CTCF and YY1 are involved in the formation of a loop allowing E2 and LCR interaction and leading to the formation of epigenetically repressed chromatin in the HPV18 genome. This event leads to attenuated expression of oncoproteins in undifferentiated cells. CTCF plays a key role in this event; in fact, the HPV18 ΔCTCF genome, carrying a mutation in the CTCF binding site, shows higher H3K4me3 and reduced H3K27me3 marks in the viral enhancer and in the downstream early promoter. This is coherent with reduced recruitment of polycomb repressor complex 2 (PCR2), enrichment of RNA pol II binding, and a consequent increase in the transcriptional activity of the viral early promoter. Pentland et al. (176) also showed that both CTCF and YY1 are necessary to repress viral transcription. During cell differentiation, a drop in YY1 expression levels leads to loss of chromatin loop formation and to increased expression of the oncoproteins consecutive to epigenetic depression of the viral genome (176).

## Transforming Activities of PVs

Studies performed on mucosal HR HPV types, such as HPV16 and 18, showed that both oncoproteins E6 and E7 play a key role in cervical cancer by altering pathways involved in the host immune response to establish a persistent infection and promote cellular transformation. The E6 (150 amino acids) and E7 (100 amino acids) oncoproteins play a central role in carcinogenesis by interacting with a large number of cellular proteins involved in key cellular events, such as the cell cycle and apoptosis control. In addition to E6 and E7, HR HPV types encode a small hydrophobic oncoprotein of < 90 amino acids, E5, which also appears to play a role in HPV-induced carcinogenesis. Continuous expression of the E6 and E7 oncoproteins is essential to initiate and to maintain the cellular transformation process. Indeed, the use of different strategies to inhibit the function of E6 and E7 in HPV-positive cancer cells resulted in cell growth arrest by apoptosis or senescence (177, 178). The main targets of the E6 and E7 oncoproteins are p53 and pRb, respectively; p53 regulates DNA damage response and apoptosis, and pRb tightly controls the cell cycle. Although E6 and E7 of mucosal HR HPV types were extensively studied, functional data on E6 and E7 of beta HPV types are sparse. Moreover, the absence of cutaneous E6 and E7 transcripts in skin tumors suggests an alternative mechanism for promoting cancer development (hit-and-run mechanism). The beta and gamma HPVs lack the E5 ORF.

### Transforming Activities of E6

The E6 oncoprotein of HPV16 is a small protein of ~150 amino acids, made up of two zinc-like fingers joined by an interdomain linker (179). E6 of HR HPV types has the ability to bind to the LXXLL peptide motif of cellular proteins. Among them, the E6-associated protein (E6AP) is an E3 ubiquitin ligase that targets proteins for ubiquitination and degradation by the proteasome (180). The best-characterized E6/E6AP interaction is with the p53 tumor suppressor (181). In normal cells, p53 plays a key role to safeguard the integrity of the cellular genome, avoiding the proliferation of cells with damaged DNA. Therefore, upon DNA damage insults, p53 will either induce cell-cycle arrest at the G1 phase to allow DNA repair or, if the DNA damage is too extensive, activate apoptotic pathways. E6/E6AP interaction leads to conformational changes of E6 that allow the association with the p53 pro-apoptotic tumor suppressor (182), leading to its ubiquitin-mediated proteasome degradation and, as a consequence, to accumulation of DNA damage and to genomic instability (183).

Although E6 of the β3 HPV49 type also showed the ability to degrade p53 by an E6AP-dependent mechanism, E6s of HPV species β2 do not share this property and have evolved alternative mechanisms to counteract p53 functions [reviewed in (19)]. For example, E6 of HPV23 interferes with the ability of the homeodomain-interacting protein kinase 2 (HIPK2) to phosphorylate p53 on serine 46 to activate its apoptotic function upon UVB irradiation (184).

In addition to inducing its degradation, E6 of the mucosal HR HPV types can inactivate p53 and abolish its transactivation *in vivo* (185), by targeting its transcriptional coactivator CBP/p300. This event leads to the displacement of p53 from CBP (186) and to the inhibition of p300-dependent p53 acetylation (187).

As for HR HPV types, the E6 oncoproteins of β1 HPV types (HPV5, 8) also have the ability to interact with p300 with high efficiency (188). HPV5 and 8 E6s inhibit the association of AKT with the p300 C-terminus that is needed to ensure p300 stability, thus leading to its proteasomal degradation (188). The degradation of p300 results in decreased protein levels of ATR, a PI3 kinase family member, which plays a key role in UV radiation damage signaling. This event in turn reduces ATR's ability to protect cells against UVB radiation-induced damage (189). The reduced ATR level results in decreased phosphorylation and subsequent attenuated accumulation of p53 in cells in response to UVB radiation exposure. In addition, in HPV5, 8, and 38 E6-expressing cells, arrest in the G1 phase of the cell cycle is prevented, which hampers DNA repair. Both events lead to increased persistence of thymine dimers and UVB radiation-induced double-strand breaks in these cells (189). More recently, Hufbauer et al. (190) showed a lack of phosphorylation of ATM, ATR, and Chk1 in HPV8 E6 expressing-monolayer and organotypic cultures leading to the impairment of DNA damage sensing and repair. Consistent with impaired activation of the ATM/ATR pathway, immunohistochemical analysis revealed the presence of thymine dimers in UVB radiation-treated E6-expressing cells (190), further confirming a role of beta HPV E6 proteins in facilitating UV radiation-induced DNA damage.

E6 of HPV8 may also play a role in skin carcinogenesis by down-regulating CCAAT/enhancer-binding protein α (C/EBPα), a transcription factor with a role in cell differentiation. MicroRNA-203 (miR-203), previously shown to be an important regulator of epidermal proliferation and differentiation, is induced by C/EBPα. In HPV8 E6 expressing keratinocytes, downregulation of C/EBPα via E6-mediated p300 degradation led to loss of miR-203 expression and induction of the ΔNP63α protein, altering the proliferation abilities of the infected epithelial cells (191).

Similar to E6s of HPV species β1, E6 of HPV38 (species β2) was able to prevent p300-mediated acetylation of p53, an event that resulted in its inhibition and thus led to the immortalization of primary keratinocytes (192), indicating that the ability of E6 proteins of HR cutaneous HPVs to induce changes in p53 acetylation status is crucial for the transforming ability of these viruses (192).

E6 has additional cellular targets that play an important role in apoptosis (e.g., Bak, survivin, TNF R1, FADD, procaspase 8) (193–197).

The anti-apoptotic protein Bak, a member of the Bcl-2 family of proteins, plays an important role in regulating the apoptotic process by forming pores with Bax to permeabilize the outer mitochondrial membrane. Bak has been shown to be targeted for degradation by different HPVs from the alpha and beta genera, an event that contributes to preventing UV radiation-induced apoptosis. The ability of the E6 proteins of beta HPVs (e.g., HPV5, 8, or 38) to target Bak for degradation, is important for promoting the survival of DNA-damaged cells and therefore for the progression of NMSC. Bak interacts with the ubiquitin ligase E6AP; however, E6 proteins of HPV5 or 8 that do not interact with E6AP (198) can still induce Bak degradation. Moreover, HPV5 E6 mediates Bak proteolysis when expressed in E6AP-null mouse embryo fibroblast (MEFs) (199), indicating that HPV5 degrades Bak via an E6AP-independent mechanism. Holloway et al. (200) have shown that HERC1 ubiquitin ligase is required for HPV5 E6-mediated Bak degradation (200). In response to UV irradiation, Bak is dephosphorylated on K113 and interacts with HERC1. The association of HERC1 and Bak is dependent upon E6 expression. In fact, HERC1 is recruited by HPV5 E6 to degrade Bak upon UV radiation-induced damage. The degradation of Bak by E6 prevents the release of proapoptotic factors including apoptosis-inducing factor (AIF) from the mitochondria into the nucleus, an event that prevents cells from undergoing UV radiation-induced apoptosis (201).

Those experiments highlight the ability of beta HPVs to enhance UV radiation-induced DNA damage, supporting a role of beta HPVs at the early stage of skin carcinogenesis.

Beta HPVs display additional features for increasing the oncogenic potential of UV radiation exposure by attenuating DNA damage repair. For instance, the binding and destabilizing of p300 by beta HPV5 and 8 E6s also lead to lower levels of BRCA1 and BRCA2, critical to the homology-dependent repair of double-strand breaks (202). Viarisio et al. (104, 121) reported a strong reduction of IL-18 production in HPV38 transgenic mice exposed to UV radiation (104, 121). IL-18 reduces UV radiation-induced DNA damage by the induction of DNA repair (203), suggesting that by inducing a reduction of IL-18 secretion in the mouse skin, HPV38 early proteins may repress the UV radiation-induced inflammasome response, favoring DNA damage accumulation and therefore promoting the carcinogenic process (104).

All HR HPV E6 proteins contain C-terminal PDZ domain-binding motifs (PBMs) that enable interaction with several cellular PDZ domain-containing proteins involved in the regulation of different processes, such as cell polarity, migration, and attachment (204). The interaction of HR HPV E6 with the PDZ proteins leads to their proteasome-mediated degradation (205). The PBM is absent from E6s of LR HPV types, suggesting a key role for this domain in HR HPV-induced carcinogenesis.

A phosphor-acceptor site for protein kinase A (PKA) is located within the PBM. Phosphorylation at this site negatively regulates the PDZ domain-binding activity. The PKA recognition sequence is highly conserved among E6 of HR HPV types and is not present in LR HPV E6 oncoproteins, further suggesting that the PDZ domain-binding activity is crucial in HR HPV-mediated transformation (206, 207). In addition, phosphorylation at this site is required for HPV18 E6 to interact with 14-3-3ξ, contributing to the stabilization of the oncoprotein (208).

Different PDZ domain-containing proteins have been identified as targets of E6 of HR HPV types: DLG1, a human homolog of *Drosophila* discs large 1, and hScrib, a homolog of the *Drosophila* scribble protein (209–211), which play an important role in the polarity of the epithelial cells and have been identified as tumor suppressors (212); the molecular scaffolds membrane-associated guanylate kinase homology proteins, such as MAGI-1 (213), which is involved in the modulation of epithelial cell adhesion and tight junction integrity, acts as a tumor suppressor through the stabilization of PTEN (214, 215); and another HPV16 PDZ domain-containing protein, CAL, which is involved in the regulation of intracellular vesicular trafficking (216). The multi-PDZ domain protein MUPP1, which negatively regulates cellular proliferation, is targeted for degradation by HPV18 E6 and possibly by other HR HPV E6 oncoproteins, thus contributing to the transforming activities of E6 oncoproteins (217). The PDZ domain-containing proteins PSD95, TIP-2/GIPC, and NHERF1, which are involved in different signaling pathways, are also targeted by E6 (218–220).

In addition to playing a role in many cellular processes, the PBM of E6 proteins also regulates the HPV life cycle; mutations of this motif were shown to lead to lower levels of episomal HPV genome and cell growth (221). HPV8 E6, which lacks the PBM, can target the PDZ domain-containing protein syntenin 2, by reducing its expression level in a MAML1-independent manner, by an epigenetic mechanism. This event plays a key role in hampering differentiation of HPV8-expressing keratinocytes (222) and provides a further example of how skin and mucosal HPV types evolved different mechanisms to alter normal cell homeostasis and induce cellular transformation.

Using a proteomic approach, Thomas et al. (223) compared the ability of E6 PBMs of HPV types with different oncogenic potentials to interact with PDZ substrates (223). This comprehensive analysis showed that the number of PDZ substrates varies according to the HPV type. Most of the PDZ substrates were recognized by HR types (i.e., HPV16 and 18), whereas HPV66, which is a possible HR HPV type, recognizes only a limited number of substrates. Of note, whereas PDZ substrates such as DLG1 are common targets of the E6 proteins irrespective of their oncogenic potential, the ability of E6 PBMs to interact with hScrib, a component of the Scribble polarity complex, is correlated with the transforming ability of the studied HPV types (223).

Telomerase activity also plays an important role during cervical carcinogenesis, because it increases with the grade of the cervical lesions (224). The ability to activate the promoter of hTERT (human telomerase reverse transcriptase), the catalytic subunit of the telomerase, by different HPVs was assessed using a luciferase-based assay. The results of that study showed that several HR HPV types significantly upregulated hTERT promoter activity (225). The induction of hTERT requires the E6/E6AP complex to interact with the heterodimer c-Myc/Max (226–229). Moreover, E6 recruits histone acetyltransferase (HAT) at the hTERT promoter, which facilitates the access of transcriptional activators to key regulatory sites through the opening of the chromatin (230).

NFX1-91, a constitutive hTERT repressor, is targeted by E6/E6AP for ubiquitin-dependent degradation (230). The induction of hTERT expression by E6/E6AP is also dependent on the activation of NFX1-123, which constitutes a level of post-transcriptional regulation (231). Together, these events lead to extending the life span of primary human keratinocytes, and to their immortalization. Proteomics studies comparing the cellular protein-binding partners of the E6s of HPV types of different genera have shown that whereas all the studied alpha HPV E6s have a high affinity for E6AP and HERC2 (232), beta and gamma HPV E6s specifically interact with the cellular transcriptional co-activator MAML1 (233). The latter interaction is required to inhibit Notch1, a key player in skin differentiation and in the activation of transcription of the cell-cycle inhibitor p21. Interaction of beta HPV E6 with the Notch pathway appears to be important to promote the transformation process of the infected keratinocytes (234, 235). The different affinities of E6s of different genera for specific cellular targets could be a consequence of their tropism and co-evolution with different type of tissues (236).

### Transforming Activities of E7

The E7 oncoprotein is made up of ~100 amino acids and has a C-terminal zinc-binding domain. HPV E7 presents three conserved regions, homologous to adenovirus E1A: CR1, CR2 in the N-terminal part, and CR3 in the C-terminal part. Moreover, HPV E7 comprises two nuclear localization sequences (NLSs) and one nuclear export sequence (NES), which enable E7 to be located in both the nuclear and cytoplasmic compartments, where E7 has different functions (237).

The CR1 domain has an UBR4/p600 binding site; the 600-kDa pRb-associated factor p600 is required for membrane morphogenesis, anchorage-independent growth, and cellular transformation (238, 239). Moreover, the interaction between BPV-1 E7 and p600 inhibits apoptosis, contributing to viral-induced transformation (240). UBR4/p600 ubiquitin ligase also appears to be involved in E7-mediated proteasomal degradation of the potential tumor suppressor PTPN14 (241), identified as a target of HR HPV E7 in cervical tumors (241, 242). The ability of HPV16 E7 to bind to p600 correlates with its capacity to transform cells (238). Similarly to E6, E7 binds to CBP/p300 and to its associated factor (pCAF) by interacting with the CBP TAZ2 domain (also known as CH3). The E7 binding site responsible for its interaction with the CBP TAZ2 domain overlaps with the LXCXE motif, which is crucial for E7 to bind to pRb. Formation of the ternary complex of E7/pRb and pCAF leads to the acetylation of pRb, an event that appears to play an important role in HPV-mediated transformation, probably by stabilizing the E7/pRb interaction (243, 244).

The CR2 domain comprises serine residues at positions 31 and 32 that are susceptible to phosphorylation by casein kinase II (CKII). Phosphorylation at these sites contributes to modulating some of the E7 functions. For example, CKII phosphorylation of E7 increases the binding affinity of HPV16 E7 for the TATA box-binding protein (TBP) (245), and it is required for efficient transformation by E7 (246). Interestingly, an additional phosphorylation site at serine position 29 exists in a natural E7 variant (N29S) and leads to increased levels of phosphorylation by CKII, which increases the interaction of E7 with TBP and pRb, and its transforming activity in primary rodent cells (247).

CR2 also comprises a conserved LXCXE motif located at residues 22–26 in HPV16 E7. This motif is essential for the interaction with pRb and related pocket proteins (p107 and p130) (248). An intact LXCXE motif is needed to induce cellular DNA synthesis and mediates cell transformation of immortalized NIH3T3 cells. Residues in the CR3 domain of E7 also seem to be required for its interaction with pRb (249). pRb and related pocket proteins (p107 and p130) play a key role in the regulation of the cell cycle by binding to and inhibiting E2F transcription factors (E2Fs), which regulates the expression of genes required for DNA synthesis and cell division. HR HPV E7 oncoproteins are able to target pRb and other members of the retinoblastoma family for proteasome-mediated degradation. This event requires the recruitment of the cullin-2 ubiquitin ligase complex, which binds to the CR1 region (250). Upon pRb interaction with HPV E7, E2F is released, with subsequent transcriptional induction of G1–S cell-cycle genes, such as cyclin A and cyclin E. The latter products are required for the activation of the cyclin-dependent kinase (CDK) complexes, which force the entry of HPV-infected cells into the S phase (251). This event leads to unscheduled growth and accumulation of genomic instability. LR HPV E7s bind to but are not efficient in targeting pRb for degradation (252). The ability of E7 to interact with pRb is not sufficient for HPV-induced transformation, which, as mentioned above, requires the interaction of E7 with additional cellular proteins (253).

The functionally and structurally pRb-related proteins, p107 and p130, are also targeted by the HPV E7 via the LXCXE motif, and both have the ability to bind to and inactivate E2Fs. These proteins are important negative regulators of the cell cycle, and they are believed to also act as tumor suppressors. However, unlike pRb, their role as tumor suppressors is still controversial, because related genetic alterations are not frequent in human cancers. Both HR and LR HPV E7s target p130 for proteasomal degradation, most likely by different mechanisms, thus highlighting an important role of this pocket protein in the HPV life cycle (254).

Analysis of HPV E7-interacting proteins showed that HPV16 E7 interacts with E2F6, which has the particularity of lacking the binding domain for pRb and acts as a transcriptional repressor. The association of HPV16 E7 with E2F6 abrogates its repressive activity on target genes, which results in increased DNA replication of HPV16-infected cells (255).

Using an organotypic culture of skin, Akgül et al. (256) showed that beta HPV8 E7-transduced keratinocytes displayed an invasive phenotype, in addition to losing their polarity (256). HPV8 E7-transduced keratinocytes also showed enhanced production of extracellular proteinases (MMP-1, MMP-8, and MT1-MMP) that play a role in the degradation of components of the basement membrane and the ECM during cell invasion. In another study, the same group used a transgenic mouse model expressing the E7 oncoprotein of HPV8 in the epidermis to investigate the molecular basis for HPV-induced invasion of skin keratinocytes. HPV8 E7 expression in mouse skin led to a reduction of E-cadherin and a subsequent upregulation of N-cadherin, an event that causes epithelial–mesenchymal transition. E7-positive keratinocytes also showed increased fibronectin expression and secretion. In addition, HPV8 E7-expressing cell surface showed enhanced levels of the integrin α3 chain, which plays a crucial role at the pre-invasive stage (257). E7 of beta-HPVs may play an important role in promoting an invasive phenotype in keratinocytes and may provide additional evidence to support a role of cutaneous HPVs in skin carcinogenesis.

HPV8 also has the ability to maintain cells in a “stemness-like” phenotype. Using *in vitro* colony formation and tumor sphere assays, Hufbauer et al. (258) showed that beta HPVs are able to increase the number of stem cell-like cells, as determined by measuring the number of HPV8 E2-, E6-, and E7-expressing cells harboring high levels of epithelial stemness cell surface markers, such as CD44 and epithelial cell adhesion molecule (EpCAM). EpCAM induction was also observed in organotypic skin cultures of HPV5, 8, and 16 E7-expressing cells, as well as in skin lesions of patients with EV (258). E7 expression also led to a reduction of the epithelial differentiation marker calgranulin B. Together, these results highlight a possible role of beta HPVs in skin carcinogenesis by increasing the number of stem cell-like cells present during early carcinogenesis and delaying cell differentiation.

Lanfredini et al. (259) evaluated KSC populations located in different regions of the hair follicle (259). In that study, using HPV8 transgenic (HPV8tg) mice that express the entire HPV8 early region, the authors identified KSCs from the upper part of the junctional zone as the site from which the expansion to the interfollicular epidermis takes place during the initial step of skin carcinogenesis. This population of stem cells express leucine-rich repeats and immunoglobulin-like domains protein 1 (Lrig-1) and are characterized by nuclear staining for the stemness marker p63. Skin accumulation of the p63 isoform, ΔNp63, which is induced by the expression of HPV8 early proteins, promotes the proliferation and expansion of Lrig1-positive KSCs. In healthy skin, the ΔNP63α transcript is directly targeted by miR-203, an event that plays a major role in the regulation of epidermal proliferation and differentiation, leading to “stemness” loss (260, 261). Of note, the skin lesions of patients with EV show strong p63 immunostaining associated with very low levels of miR-203.

In addition to features cited above, E7 can also deregulate the cell cycle by binding and abrogating the inhibitory effect of CDK inhibitors like p21^Cip1^ and p27^Kip1^. This interaction abolishes their inhibitory effect on the cell cycle and highlights a different mechanism by which E7 favors cell proliferation (262–264).

E6 and E7 of HPV38 and 49, which can efficiently immortalize human primary keratinocytes, do not have the ability to target pRb for degradation when expressed in these cells (120). However, similarly to E7 of LR mucosal types HPV38 and 49 E7s can efficiently bind to pRb. In contrast, beta HPV8 E7 binds to pRb to a lesser extent compared with HPV16 E7, but it is still able to repress pRb levels when expressed in keratinocytes (265, 266).

Studies from our laboratory have shown that different beta HPV types (e.g., HPV38) are able to trigger pRb hyperphosphorylation, an event that leads to release of E2F and to the transcriptional activation of E2F target genes [reviewed in (19)]. In addition, E7 of HPV38 shows the ability to counteract p53-mediated apoptosis by inducing accumulation of the p73 isoform, ΔNp73, by two different mechanisms: (i) by inducing the recruitment of a post-translationally modified form of p53 to the promoter of ΔNp73, which activates its transcription (267), and (ii) by mediating ΔNp73 protein stabilization with an IκB kinase beta (IKKβ)-mediated mechanism (268). The latter studies highlight that cutaneous HPVs have evolved different mechanisms than HR mucosal HPVs, to target key cellular proteins (pRb and p53) and promote cellular transformation.

### Transforming Activities of E5

Although the transforming activities of E6 and E7 oncoproteins are well characterized, the role of the E5 oncoprotein is still poorly understood (269). E5 is a hydrophobic membrane-associated protein that localizes to the endoplasmic reticulum, Golgi apparatus, and plasma membrane (270, 271). E5 forms a hexameric viroporin complex, which modulates ion homeostasis (269). E5 viroporin is required for the hyperactivation of mitotic signaling, and it plays a key role during the life cycle of HR HPV types. Recombinant HPV16 E5 membrane channel activity is sensitive to compounds such as adamantine and rimantadine. Inhibition of E5 viroporin activity in primary human keratinocytes harboring HPV18 genome leads to decreased ERK-MAPK phosphorylation and cyclin B1 levels, and to an increase in the expression of differentiation marker (272). E5 viroporin activity is therefore critical for maintaining mitogenic signaling and delaying expression of differentiation marker during the productive stages of the HPV18 life cycle; therefore, targeting viroporin could pave the way for an effective antiviral strategy.

The expression of E5 increased the immortalization effects of HPV16 E6 and E7 (273). E5 is involved in the transformation of primary human keratinocytes by potentiating epidermal growth factor receptor (EGFR) signaling (274). In 16E5 transgenic mice, EGFR is required for epithelial hyperplasia (275). The activation of EGFR leads to induction of the expression of cyclooxygenase-2 (COX-2), which plays an important role in cervical carcinogenesis, through nuclear factor-kappa B (NF-κB) and AP-1 (276). Via stimulation of EGFR, HPV16 E5 induces Met, a growth factor receptor involved in tumor cell motility and cancer metastasis; moreover, Met signaling is required for proper differentiation-dependent viral gene expression (277).

HPV16 E5 protein enhances, in cooperation with EGFR signaling, the down-regulation of tumor suppressor p27^Kip1^ levels, which promotes cell-cycle progression (278). The level of another CDK inhibitor, p21, is reduced in cells that over-express E5 (279). Both events lead to an increase in cell proliferation and in the percentage of cells in the S phase.

HPV16 E5 also contributes to carcinogenesis by inhibiting apoptosis by multiple molecular strategies. The effect of HPV16 E5 on apoptosis was assessed using raft cultures in which E5 prevented FasL- or TRAIL-mediated apoptosis (280). A recent study reported that HPV16 E5 specifically down-regulates miR-196a in cervical cancer cells. The same study showed that miR-196a expression affects cell proliferation, cell growth, and apoptosis, thus confirming the role of HPV16 E5 in cervical cancer development (281). Another anti-apoptotic strategy consists of decreasing pro-apoptotic Bak and Bax levels, and increasing the expression of the anti-apoptotic Bcl-2. HPV16 E5 inhibits apoptosis of cervical cancer cells by stimulating the ubiquitin-proteasome-mediated degradation of Bax protein (282). It has also been reported that HPV16 E5 protects E5-expressing foreskin keratinocytes from UVB radiation-induced apoptosis (283).

All these data indicate that expression of E5 contributes to HPV-driven cellular transformation. However, unlike E6 and E7, E5 is not required for the maintenance of the oncogenic phenotype, because the E5 ORF is frequently disrupted during HPV integration, thus rather highlighting a role of E5 at the initial step of carcinogenesis (284).

HPV types from different genera (beta, gamma, and mu) lack the E5 ORF, suggesting that the corresponding functions are performed by other viral proteins.

E5 forms a complex with EVER1 and EVER2, proteins encoded by the TMC6 and TMC8 genes, respectively, (92). Most of the patients with EV who are susceptible to persistent beta HPV infections carry mutations in these genes. The EVER proteins interact with the zinc transporter ZnT-1 in order to regulate intracellular zinc homeostasis. Zinc sequestration by the ZnT-1/EVER complex may inhibit the replication of EV-associated HPV types. In contrast, mutations in EVER proteins lead to the disruption of the ZnT-1/EVER complex, and an increase in the level of zinc in the cytoplasm, resulting in increased replication of EV-associated HPV types (285), [reviewed in (286)]. A recent study reported that patients with EV can also display the typical EV phenotype, bearing non-synonymous calcium- and integrin-binding protein 1 (CIB1) mutations and lacking TMC6 and TMC8 mutations (287). CIB1 does not seem to interact with ZnT-1, but this protein forms a complex with EVER1 and EVER2. The CIB1-EVER1-EVER2 complex appears to act as a restriction factor for infection by cutaneous HPVs that do not express E5. In patients with EV, impairment of CIB1, EVER1, or EVER2 allows replication of these HPV types and development of EV lesions on the skin (287).

## Conclusive Remarks, Challenges, and Open Questions

In the past decades, epidemiological and biological studies have clearly demonstrated the role of mucosal HR HPV types in human carcinogenesis. While co-evolving with the human host, HPVs have developed strategies to evade the immune system and create the optimal conditions to persist in the host for many years. During HPV chronic infection, the E6 and E7 oncoproteins interact with different cellular proteins to prevent apoptosis, counteract the cellular senescence program, and promote unscheduled cell growth. These activities of HR HPV E6 and E7 are part of a viral strategy to complete the viral life cycle and generate progeny. The expression of the oncoproteins is kept under the tight control of E2. Loss of E2 repressive functions, by viral integration or by other mechanisms, which are discussed in this review, leads to constitutive high expression of E6 and E7, a key event in HPV-mediated transformation (Figure 3). Although the mechanisms leading to cervical cancer have been well-characterized, there is still a gap in the understanding of the pathogenesis of other HPV-related malignancies, e.g., oropharyngeal cancer. The proportion of HPV-driven oropharyngeal cancers has been increasing over the past decades in many parts of the world, mainly in Europe and North America (288–290). The natural history of HPV infection in the oral cavity and oropharynx is poorly studied and needs further investigation.

Of note, recent studies identified HPV16 E6 antibody as a good diagnostic and prognostic marker of oropharyngeal cancer. HPV16 E6 seropositivity was detected more than 10 years before the diagnosis (291, 292).

Moreover, although the available prophylactic vaccines have demonstrated their effectiveness in the context of prevention of anogenital cancers (293), very limited epidemiological data are available on the efficacy of the HPV vaccine on oral HPV infection (294). This deserves further investigation.

Prophylactic vaccines show a good efficiency in covering targeted HPVs, but they are inefficient in existing infections. Therefore, therapeutic vaccines are needed to fill this gap. Several studies have reported the development of promising therapeutic vaccines. Their use in the near future could be expected; however, their cost could be high. This limitation could be overcome by the use of inexpensive technologies (i.e., plant-made therapeutics) (295–299).

One of the most recurrent questions regarding the prophylactic vaccine is whether HPV vaccination will result in the occupation of a newly vacant ecological niche by non-targeted HPV types. This hypothesis has been referred as “type replacement. ” To date, the available data do not support this hypothesis. However, as reported in recent studies, a few types (i.e., HPV51 and 52) may need to be monitored after vaccination (300, 301).

The bacterial microbiome composition may also play a role in the outcome of mucosal HPV infection. A chronic mycoplasma infection was shown to promote cervical dysplasia induced by HPV (302). Therefore, identifying the bacterial microbiome could allow the identification of patients at high risk for developing cervical cancer.

Our knowledge of the biology of beta and gamma HPV types is still uncomplete. For example, the expression patterns of cutaneous HPV types and associated splicing patterns are largely unknown, except for HPV5 and 8 (https://pave.niaid.nih.gov/). There are also important gaps in the understanding of the life cycle of cutaneous HPV types, which also deserve further research.

In February, 2009, an IARC Monograph Working Group met at IARC to assess the carcinogenicity of biological agents including beta and gamma HPV types. HPV5 and 8 were classified as possibly carcinogenic to humans in patients with EV (IARC Group 2B), whereas the other cutaneous HPV types were each determined to be “not classifiable as to its carcinogenicity to humans” (IARC Group 3), on the basis of insufficient epidemiological and biological data available at that time (10). In fact, although several epidemiological and biological studies support a role of β1 and β2 HPV types in NMSC, the lack of cutaneous HPV E6 and E7 expression in SCC has raised the question of whether the viral proteins are required in the carcinogenesis process.

Since that 2009 meeting, strong epidemiological and experimental data in favor of a role of cutaneous HPV types as cofactors in skin carcinogenesis have been published, as described in this review. Indeed, recent findings from our group and others have shown that unlike the expression of oncoproteins of HR HPV types, cutaneous HPV E6 and E7 expression appears to be required only at the initial step of skin carcinogenesis by exacerbating the deleterious effects of UV radiation (104). Over the past decade, studies using experimental animals (HPV38 transgenic mice and the MnPV-infected rodent model *Mastomys coucha*) have provided strong *in vivo* evidence for a synergism between UV radiation and cutaneous HPV types in the development of cSCC (104, 107). Most importantly, both models provided further evidence for a hit-and-run mechanism. However, additional studies are still necessary to validate this hypothesis in humans.

In addition to these experimental data, a recent prospective study of two organ transplant recipient cohorts showed that having five or more different beta HPV types in eyebrow hair, and a high beta HPV viral load, was associated with cSCC carcinogenesis, providing strong evidence for a role of beta HPVs in this cancer, whereas no evidence was found for basal cell carcinoma (303). The development of vaccination strategies that target cutaneous HPV infections would be of great benefit to these patients (304, 305).

Another open question regards the tropism of certain HPVs. As previously specified in this review, a dual tropism of HPV species β3 has been proposed. Moreover, this species displays biological similarities with mucosal HR HPV types (111, 120). Our recent data highlighted that E6 and E7 of HPV49 share some features with HR mucosal types, and efficiently transform epithelial cells in *in vitro* and *in vivo* models (120, 121). In addition, in line with these biological data, epidemiological studies have shown β3 HPVs and other cutaneous types in the oral cavity mucosa. Beta and gamma HPV types have also been isolated from the nasopharynx and the nasal mucosa (119, 306). Moreover, a recent study based on a prospective design reported that beta or gamma HPV types were associated with the incidence of HNC (117). Those findings warrant further studies to better understand the role of those so-called “cutaneous HPV types” in mucosotropic cancers (111, 119).

A large number of gamma HPV types have been isolated; however, their biological activities are poorly studied and deserve more investigation. Finally, some of the known but not yet biologically characterized beta or gamma HPV types may display transforming activity and also deserve attention. In addition to these not-yet-studied types, one may also consider the large number of novel HPVs found in recent years, as well as the rapid evolution of viral detection and screening techniques and large data set analysis pipelines.

For all these reasons, we believe that in the coming years the field of HPV research will continue to be a source of surprises and excitement for the scientific community.

## Author Contributions

The author confirms being the sole contributor of this work and has approved it for publication. TG wrote the manuscript and designed the figures.

### Conflict of Interest Statement

The author declares that the research was conducted in the absence of any commercial or financial relationships that could be construed as a potential conflict of interest.
